# *Gelidiella acerosa* protects against Aβ 25–35-induced toxicity and memory impairment in Swiss Albino mice: an *in vivo* report

**DOI:** 10.1080/13880209.2017.1302967

**Published:** 2017-03-21

**Authors:** Syad Arif Nisha, Kasi Pandima Devi

**Affiliations:** a Department of Biotechnology, Srimad Andavan Arts and Science College (Autonomous), Trichy, India;; b Department of Biotechnology, Alagappa University (Science Campus), Karaikudi, India

**Keywords:** Alzheimer’s disease, oxidative stress, β-secretase, apoptosis, monoamine oxidase, AChE, BuChE, toxicity, caspase-3

## Abstract

**Context:** Alzheimer’s disease (AD) is believed to develop due to deposition of β-amyloid (Aβ) peptide. Hence, efforts are being made to develop potent drug that target amyloid hypothesis.

**Objective:** The present study explores the effect of the seaweed *Gelidiella acerosa* (Forsskål) Feldmann & Hamel (Gelidiellaceae) against Aβ 25–35 peptide in Swiss albino mice.

**Materials and methods:** The animals were administered through intracerebroventricular (ICV) injection with the Aβ 25–35 peptide (10 μg/10 μL/ICV site) on 21st day of the pretreatment of *G. acerosa* (whole plant) benzene extract (200 and 400 mg/kg bw). On day 30, animals were sacrificed and brain tissue homogenate was prepared. The activities of AChE, BuChE, b-secretase, MAO-B, and caspase-3 were determined, and Bax expression was assessed by Western blotting.

**Results:***Gelidiella acerosa* benzene extract restored the level of antioxidant enzymes and prevented lipid and protein oxidation significantly (*p <* 0.05). The extract protected the mice from cholinergic deficit significantly (*p <* 0.05) by inhibiting the activities of AChE and BuChE, which was about 0.116 ± 0.0088 U/mg of protein and 0.011 ± 0.0014 U/mg of protein respectively, which was otherwise increased in peptide-treated group (0.155 ± 0.007 U/mg of protein and 0.015 ± 0.0012 U/mg of protein respectively). Interestingly, *G. acerosa* benzene extract inhibited β-secretase and MAO-B activity. Reduction (*p <* 0.05) in level of caspase-3 activity and Bax expression suggests that *G. acerosa* protects the cells from apoptosis.

**Discussion and conclusion:** The results suggest that *G. acerosa* possesses excellent neuroprotective potential against peptide mediated toxicity under *in vivo* conditions.

## Introduction

Alzheimer’s disease (AD) is a neurodegenerative disorder characterized by cognitive impairment (clinically) and by the appearance of senile plaques and neurofibrillary tangles (pathologically) (Cho et al. 2009). The amyloid beta (Aβ) peptides are the major components of senile plaques, which are derived from the altered proteolytic processing of Amyloid Precursor Protein (APP). BACE-1 (β-secretase) is the enzyme which is responsible for the generation of toxic Aβ fragments from APP, thereby it promotes the toxicity in brains of AD (Zhao et al. 2013). In addition to the presence of these neuropathological hallmarks, several neurotoxic mechanisms are involved in the disease occurrence and progression, which includes oxidative stress, cholinergic deficit, Aβ accumulation, apoptosis, neuroinflammation and neurovascular dysfunction. Since these abnormal changes occur simultaneously, which involves complex mechanisms (in AD), there is a considerable setback in the complete understanding of the cause and consequences of it. However, noticeable attempts were made to reveal the pathways that worsen the condition and to develop effective therapeutic approaches for the betterment of AD patients. The U.S. Food and Drug Administration (FDA) have approved certain drugs such as tacrine, donepezil, memantine, galanthamine and rivastigmine with the capability to inhibit cholinesterases (ChEs). Although these drugs possess therapeutic potential, all of them act on single target that is ChEs. Whereas, as the disease progresses, other neurotoxic mechanisms becomes active and causes further disease progression.

For the last several decades, the use of natural compounds has been in practice for the treatment of various diseases. Seaweeds or marine algae have provided a great biological diversity for sampling in the phase of drug discovery and development. They are not only significant sources of essential proteins, vitamins, and minerals, but several species of algae also produce or contain secondary metabolites, polysaccharides, and glycoproteins with antitumor and immuno-stimulatory activity. Marine algae contain large amounts of characteristic bioactive molecules like polysaccharides, sterols, terpenoids and fatty acids with potential therapeutic activities (Choi et al. 2009). Ryu et al. (2003) demonstrated that the farnasylacetone derivatives derived from marine brown alga, *Sargassum sagamianum* Yendo (Sargassaceae) exhibit excellent cholinesterase inhibitory activity. The sulfated polysaccharides from *Sargassum wightii* Greville ex. J. Agardh (Sargassaceae) was found to possess potential antioxidant activity, when evaluated in rats with oxidative liver injury (Josephine et al. 2008). Hence, in the present study, the multipotent neuroprotective action of the marine red macro alga, *Gelidiella acerosa* (Forsskål) Feldmann & Hamel (*Gelidiellaceae*) was investigated. *Gelidiella acerosa* has been routinely employed as the principle agarophyte for the production of superior quality of agar. Recent reports demonstrated that *G. acerosa* was found to exhibit potential antioxidant (Megha & Anjali 2013), anticancer (Duraikannu et al. 2014) and antimicrobial activities (Elsie & Dhanarajan 2010; Vivek et al. 2011), when evaluated through various *in vitro* and *in vivo* systems. Preliminary phytochemical screening of ethanolic extract of *G. acerosa* suggests that the seaweed contains high amount of secondary metabolites such as terpenoids, alkaloids, flavonoids and phytosterols (Elsie & Dhanarajan 2010). Research from our group demonstrated that *G. acerosa* benzene extract possess excellent antioxidant (Suganthy et al. 2013), antiChE (Syad et al. 2012) and anti-amyloidogenic activities (Syad et al. 2013). Moreover, recent studies from our group revealed that bioactive-guided fractionation revealed the presence of diterpene, phytol, which was further confirmed through molecular docking studies (Syad et al. 2016). With this background information, in the present study, the neuroprotective effect of *G. acerosa* benzene extract was evaluated on Aβ 25–35-treated albino mice.

## Materials and methods

### Animals

Healthy male Swiss albino mice (5–6 weeks of age) weighing 22–25 g were employed for the present study. The animals were housed in plastic cages in a ventilated room with 12 h cycle of day and night and temperature was maintained around 25 °C. They were fed with standard pellet diet (Hindustan Lever Ltd, Mumbai). All the procedures followed were approved by the Institutional Animal Ethics Committee of C.L. Baid Metha College of Pharmacy, Chennai, India (Protocol No: IAEC/321/06/CLBMCP/2011; dated on 22/06/2011).

### Seaweed collection and solvent extraction

The marine red alga, *G. acerosa* (50 kg) was collected in the month of July 2009 from south Indian coastal area and the species was identified according to the references Oza and Zaidu (2003) and Krishnamurthy and Joshi (1970) and further confirmed by Dr. M. Ganesan, Scientist, CSMCRI, Mandapam Camp, Tamil Nadu. The voucher specimen was deposited at Department of Biotechnology, Alagappa University, under the accession number AUDBTGA20100101. The collected seaweeds were washed thoroughly in distilled water, dried under shade, powdered and used for extraction. The powdered seaweed (100 g packed for each extraction) was subjected to solvent extraction using the solvents petroleum ether and then followed by benzene. The extract was then subjected to complete evaporation and stored in air-tight container. The yield of the extracts was 0.06%.

### Intracerebroventricular (ICV) injection of Aβ 25–35 peptide in brain and *G. acerosa* benzene extract treatment

Details regarding dosage and treatment of Aβ 25–35 peptide and *G. acerosa* benzene extract are given in Table 1. Aβ 25–35 (1 mM) was dissolved in sterile saline and allowed to aggregate by incubating at 37 °C for 4 days. ICV injection of Aβ 25–35 (10 μg/10 μL/ICV site) was performed in mice according to the procedure established by Laursen and Belknap (1986) (by identifying the bregma point) using stereotaxic apparatus. The peptide was administered to groups II, III, IV and VI on 21st day of the pretreatment of *G. acerosa* benzene extract. After the peptide administration, the extract treatment was continued for eight consecutive days. Neither the insertion of the needle nor injection of saline induced any significant changes on survival, behavioural response or cognitive functions. After the peptide injection, the animals were subjected to behavioural tests for eight days. On day 30, all the animals were sacrificed, brain were removed and used for further experimental studies.

**Table 1.** Details regarding dosage and treatment of Aβ 25–35 peptide and *G. acerosa* benzene extract in Swiss Albino mice.

Groups	Treatment	Dose
I	Vehicle control	1% Carboxymethyl cellulose (CMC) + PBS
II	Aβ 25–35 treatment	10 μM Aβ 25–35 in PBS
III	Aβ 25–35 + Extract treatment (Low dose)	10 μM Aβ 25–35 in PBS +200 mg/kg BW of *G. acerosa* benzene extract in CMC
IV	Aβ 25–35 + Extract treatment (High dose)	10 μM Aβ 25-35 in PBS +400 mg/kg BW of *G. acerosa* benzene extract in CMC
V	Extract alone	400 mg/kg BW of *G. acerosa* benzene extract in CMC
VI	Aβ 25–35 + Donepezil	10 μM Aβ 25–35 in PBS +1 mg/kg BW of donepezil in CMC
VII	Donepezil alone	1 mg/kg BW of donepezil in CMC

### Behavioural studies

#### Y-maze test

Y-maze test was performed to assess the short-term memory in mice (Maurice et al. 1996). The maze was constructed in such a way that each arm was 40 cm long, 12 cm high and 3 cm wide at the bottom and 10 cm wide at the top. The maze was wood painted in black and the arms were converged at an equilateral triangular central area, which was 4 cm at its longest axis. The entire apparatus was placed on the floor of the experimental room and was illuminated with 100 W bulb from 200 cm above. Each mouse was placed at the end of one arm and allowed to move freely in a standard dimension through the maze for about 8 min and the series of arm entries including the possible returns into the same arm was recorded visually. The ability of the mouse to remember the arm, which it had already visited, is its ability to alternate. Alternation can be defined as the number of successive entries into all the three arms on consecutive occasions. Percentage of alternation was calculated using the following formula:

% of Alternation = [(Number of alternations)/(Total arm entries – 2)] × 100

#### Water maze test

Water maze test was performed (to assess the spatial learning and memory) according to Morris (1984) with modifications. The apparatus was made of circular water tank (100 cm diameter and 35 cm height) containing water at 28 °C to a depth of 15 cm and the water was made opaque by adding titanium oxide. The water pool in the tank was divided into four equally spaced quadrants and the platform (4.5 cm diameter and 14.5 cm height) was placed in such a way that its top was submerged 2 cm below the water surface in one particular quadrant of the maze. The mice were not allowed to swim in the pool before training. During the training trials, the time required to escape into the hidden platform in one of the quadrant was recorded. The escape latency to reach the platform was measured in four training sessions for 4 days, corresponding to third, fourth, fifth and sixth day after ICV injections. The latencies were calculated as mean of total time spent in four trials of each day. The number of times the platform was not found was also recorded.

### Step-down inhibitory avoidance test

The step-down inhibitory avoidance test was performed to evaluate the non-spatial long-term memory. The study was performed according to the method of Sakaguchi et al. (2006) with certain modifications. The study apparatus consisted of 50 cm ×25 cm ×25 cm acrylic box whose floor was 1 cm apart. A 7 cm wide, 2.5 cm high and 25 cm long platform occupied the centre floor. During the training sessions (on the seventh day after ICV injection), the animals after stepping down, they were allowed to place their paws on the grid, which received 0.4 mA scrambled foot shock for 2 s. In the test session (on the eighth day after ICV injection), no foot shock was given and step-down latency was used as a measure of retention of memory. One-trial step-down inhibitory avoidance involves the activation of two separate memory types, a short-term memory (STM) and long-term memory (LTM) system. Hence, the retention tests were carried out for 90 min (STM) and 7 days (LTM). The same mice were used for both the tests, as testing for STM did not affect LTM scores. Each mouse was placed again on the platform and the step-down transfer latency time was recorded.

### Preparation of tissue homogenate

After completing the behavioural tests, all the animals were sacrificed and the whole brains were collected and subjected to homogenization. The homogenate was prepared using 100 mM phosphate buffer (pH 7.4) containing 1% Triton X-100. Centrifugation was performed at 5000 rpm for 20 min at 4 °C. The supernatant was collected separately and stored at −20 °C and used for all the biochemical assays. Protein estimation was done for all the samples using the method followed by Lowry et al. (1951).

### Evaluation of antioxidant potential of *G. acerosa* benzene extract

The antioxidant potential of *G. acerosa* benzene extract was assessed by monitoring the levels of endogenous antioxidant defence systems in mice brain tissue homogenate. The activity of the antioxidants such as catalase, SOD, GSH, GR, GPx and GST were evaluated according to the methods of Aebi (1984), Paoletti and Mocali (1990), Sedlak and Lindsay (1968), Carlberg and Mannervik (1975), Vernet et al. (1999) and Habig et al. (1974), respectively.

### Macromolecular damage assessment in mice (treated with Aβ 25–35 and/or extract) brain tissue homogenate

#### Lipid peroxidation assay

Measurement of thiobarbituric acid reactive substances (TBARS) content is a well-recognized and established method for the quantification of lipid peroxides (Yagi & Rastogi 1979). Brain tissue homogenates were mixed with 0.37% tribromoacetic acid (TBA) and 15% TCA (prepared in 0.25 N HCl). The reaction mixture was heated for 15 min at 100 °C and then cooled at room temperature. Centrifugation was performed at 3000 rpm for 15 min and the supernatant was collected and the absorbance was measured at 532 nm. The results were expressed as μM of TBARS/mg of protein.

#### Measurement of protein carbonyl content

Protein carbonyls have been considered as excellent markers for protein oxidation (Levine et al. 1990). The amount of protein carbonyls present in the biological system can be conveniently detected using dinitrophenyl hydrazine (DNPH) method is a widely used and convenient method for detection of protein carbonyls in biological systems. Protein carbonyls readily react with DNPH and forms corresponding Schiff’s base to produce corresponding hydrazone, which can be analyzed spectrophotometrically. Brain tissue homogenates from different experimental groups were treated with equal volume of 0.2% DNPH (prepared in 2.5 N HCl) and incubated in dark for 1 h. The samples were then precipitated with 10% TCA and centrifuged at 3500 rpm for 20 min. The precipitate was washed with ethanol-ethyl acetate (1:1 v/v). The samples were then centrifuged at 3500 rpm for 20 min. The pellet was finally suspended in 2 mL of 2 M guanidine HCl and incubated for 10 min at 37 °C. The amount of protein carbonyl present was measured at 370 nm. The results were expressed as mM of free carbonyl content/mg of protein.

### Cholinesterase inhibitory assay

AChE and BuChE inhibitory activity was evaluated by Ellman et al. (1961) and Ingkaninan et al. (2000). The AChE and BuChE reacts with the substrates ATCI and BTCI (respectively) results in the formation of thiocholine iodide, which in turn reacts with DTNB and forms 5-thio-2-nitrobenzoate. The formation of this product can be determined by measuring the absorbance at 405 nm. Tissue homogenates from different experimental groups were mixed with 1 mM DTNB and the total volume was made up to 1 mL with Tris-HCl buffer (pH 8.0). 1 mM acetylthiocholine iodide (ATCI) or butyrylthiocholine iodide (BuTCI) was added to initiate the enzyme activity. The formation of 5-thio-2-nitrobenzoate anion was detected by yellow colouration and the absorbance was detected in the wavelength of 405 nm. The specific activity of the enzyme was determined for all the control and test samples.

### Measurement of caspase-3 activity

The level of caspase-3 activity was measured in the tissue homogenates of all the experimental groups (Ochu et al. 1998). The assay is based on the hydrolysis of acetyl-Asp-Glu-Val-Asp p-nitroanilide (Ac-DEVD-pNA) by caspase-3, which results in the release of p-nitroanilide (pNA). The amount of pNA released from the substrate is calculated from the absorbance values at 405 nm. In order to determine the caspase-3 activity, tissue homogenate was prepared using a specific homogenizing buffer, which consisted of 50 mM Tris-Cl (pH 7.4), 1 mM EDTA, 10 mM EGTA, 1 mM DTT and 0.1 M phenazine methosulfate. The tissue sample in buffer solution was centrifuged at 12,000 *g* at 4 °C for 20 min. Protein estimation was done for the homogenate by the method of Lowry et al. (1951). The homogenate was used as the enzyme source and the enzyme activity was calculated from the standard curve plotted using *p*-nitroanilide (25–200 μM). The results are expressed as Units/mg of protein.

### β-Secretase assay

β-Secretase (BACE-1) activity was assessed by BACE-1 activity assay kit (Biovision, Milpitas, CA). The β-secretase activity was measured using 96-well (Nunc F16 Black MaxiSorp TM) black polystyrene microplate. The brain tissues obtained from the experimental animal were used for the preparation of homogenate. Ice-cold homogenizing buffer was added to the tissue samples and incubated in ice for 10 min. The mixture was centrifuged at 10,000 *g* for 5 min and the supernatant was collected separately and stored. Brain tissue homogenate (50 μL) from different experimental groups were added separately to each well of 96-well plate. Active β-secretase (2 μl) was used as positive control and was added to 50 μL of extraction buffer. β-Secretase inhibitor (2 μL) was added to the sample and was considered as negative control. Reaction buffer (50 μL) was added to all the wells and incubated for 5–10 min at 37 °C for 1 h. After incubation, the fluorescent intensity was measured using a multilabel reader (Molecular Device Spectramax M3 Sunnyvale, CA, USA, equipped with Softmax Pro V5 5.4.1 software) with the excitation and emission wavelength of 335 and 495 nm respectively. The β-secretase activity was expressed as relative fluorescence units (RFU)/μg of protein.

### Determination of monoamine oxidase B (MAO B) activity

The method involves the use of kynuramine, which is a common substrate for MAO B. The enzyme catalyzes the spontaneous cyclization of the intermediate aldehyde formed by the oxidative deamination of kynuramine to form 4-hydroxyquinoline as the end product. The formation of 4-hydroxyquinoline can be measured fluorimetrically (Krajl 1965). Brain tissue homogenates from different experimental groups were incubated with 1 μM of clorgyline (MAO A inhibitor) for 15 min. After incubation, 750 μL of phosphate buffer (pH 7.4) and 200 μL of kynuramine (500 μM) was added and incubated at 37 °C for 30 min. The reaction was terminated by the addition of 10% ZnSO4 (250 μL), 1 N NaOH (50 μL) and centrifuged at 3000 rpm for 5 min and the supernatant was collected separately. Supernatant (0.7 mL) was mixed with 1.4 mL of 1 N NaOH and the fluorescent intensity was measured at the excitation wavelength of 315 nm and emission wavelength of 380 nm using fluorescence spectrometer. The amount of 4-hydroxyquinoline formed was determined from the standard curve plotted using kynurenic acid (25–100 μM). The results were expressed as U/mg of protein (1 Unit = nmol of 4-nitroquinoline formed/min).

### Western blot analysis

Brain tissue homogenates were used for western blot analysis in which the protein concentration was determined according to the method of Lowry et al. (1951). The proteins were separated in sodium dodecyl sulphate polyacrylamide gel and transferred to PVDF membrane. The membrane after transfer was subjected to blocking with 5% non-fat dry milk and was incubated overnight at 4 °C with primary antibodies [(Bax and BCl-2); Rabbit polyclonal Ab; Santa Cruz Biotechnology, Inc]. Then the membrane was incubated with respective secondary antibodies (Anti-Rabbit IgG labelled with alkaline phosphatase; Santa Cruz Biotechnology, Inc.). The proteins were detected using chromogenic substrate (BCIP/NBT detection system).

### Toxicity evaluation of *G. acerosa* in Swiss Albino mice

#### Acute toxicity test

Acute toxicity study was carried out as per OECD (Organization for Economic Co-operation and Development) guidelines (OECD, 423). Animals were divided into two groups (Control and extract treated) of six animals each. All the animals were deprived of food for 16 h prior to the administration of test drug. The control group received 0.1% CMC (carboxymethyl cellulose) orally, the other groups were treated with *G. acerosa* benzene extract aseptically suspended in 0.1% CMC solution and administered in a single oral dose of 2000 mg/kg of body weight by gavage. The general behaviour and presence of toxicity was observed and recorded periodically at 1, 2, 4, and 6 up to 24 h after drug administration. The presence and absence of convulsions and tremors were monitored and recorded. Presence of any abnormal clinical symptoms was also noted.

#### Sub-acute toxicity test

Sub-acute toxicity test was also performed to evaluate the toxicity of *G. acerosa* benzene extract during the repeated dosage for 28 days. The animals were divided in to three groups of six animals each. *G. acerosa* benzene extract was suspended in 0.1% CMC and the mixture was administered orally by gavage at two dose levels 200 and 400 mg/kg of body weight. The extract was administered daily for a period of 28 days. Animals in the control group were administered with highest volume of 0.1% CMC used for extract suspension. All the animals were weighed and observed daily for water and food intake. Presence of physiological and behavioural changes was also monitored.

#### Analysis of haematological and biochemical parameters

At the end of the study, whole blood sample was collected by cardiac puncture and used for haematological studies. Haematological and biochemical analysis was performed for both acute and sub-acute toxicity studies. The parameters analyzed were RBC, WBC, platelet count, packed cell volume, blood sugar, blood urea nitrogen (BUN), serum creatinine, alkaline phosphatase, serum total protein, serum albumin, serum glutamic oxaloacetic transaminase (SGOT), haemoglobin (Hb), mean corpuscular volume (MCV), mean corpuscular haemoglobin (MCH), neutrophils, lymphocytes, eosinophils, monocytes, basophils, serum glutamic pyruvic transaminase (SGPT), calcium, sodium, potassium, bilirubin, serum total cholesterol, serum triglycerides, serum HDL cholesterol, serum LDL cholesterol and serum VLDL cholesterol.

#### Histopathological analysis

Histopathological analysis was done using the vital organs like liver, brain, kidney and heart. All the organs were collected, weighed and visually inspected for pathological changes in tissues, followed by preservation in 10% formalin for histopathological analysis. The collected tissues were embedded in paraffin, sliced as thin layers, fixed in slides and subjected to haematoxylin-eosin staining. The pathological observations were performed on gross and microscopic basis.

### Statistical analysis

Experimental results concerning this study were represented as mean ± S.D. of three parallel measurements. Analysis of variance was performed by one-way ANOVA. Significant differences between means were determined by Duncan’s multiple range tests. *p-*Value* <*0.05 were regarded as significant.

## Results

### Evaluation of protective effect of *G. acerosa* benzene extract on Aβ 25–35-induced memory impairment in mice by behavioural tests

#### Assessment of protective effect of *G. acerosa* on Aβ 25–35 induced memory impairment by Y-maze test

**Figure 1.**
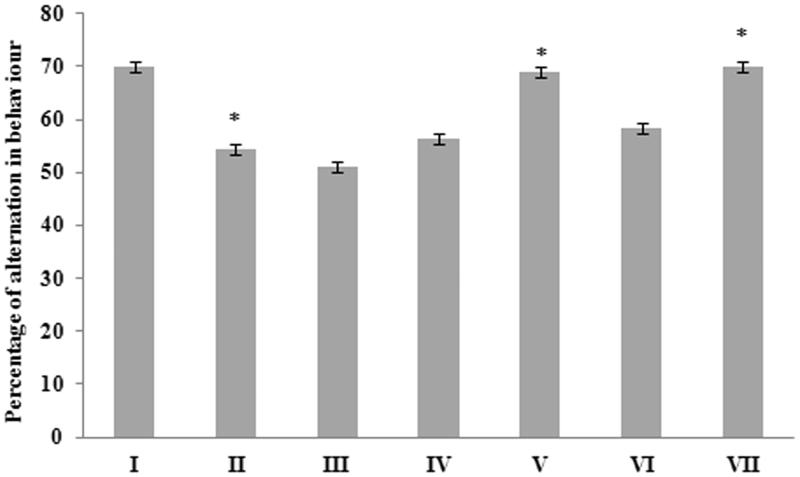
Effect of *G. acerosa* benzene extract on Aβ 25–35-induced short-term memory by Y-maze test. The results are expressed as Mean ± SD. *p < 0.05 [Comparisons were made between groups II (Aβ 25–35 peptide treated) Vs I (Carboxy methyl cellulose (CMC) treated) & III (Aβ 25–35 peptide +200 mg/kg of extract in CMC), IV (Aβ 25–35 peptide +400 mg/kg of extract in CMC), V (400 mg/kg bw of extract), VI (Aβ 25–35 peptide + donepezil), VII (1 mg/kg bw of donepezil) Vs II (Aβ 25–35 peptide treated)].

Experimental mice were examined three days after ICV administration of Aβ 25–35, for spontaneous alternation behaviour in Y-maze, which is an index of spatial working memory. Administration of Aβ 25–35 resulted in a significant (*p <* 0.05) decrease in % of alternation behaviour (54.33 ± 1.50) when compared to the control group (69.83 ± 8.08). Treatment with *G. acerosa* benzene extract resulted in an increase in alternation behaviour % (56.33 ± 1.21%) at the concentration of 400 mg/mL (Group IV) (Figure 1). Donepezil, a standard anti-Alzheimer drug did not induce any significant increase in the % of alternation behaviour in peptide-treated mice (Group VI).

#### Assessment of protective effect of *G. acerosa* on Aβ 25–35-induced memory impairment by water-maze test

**Figure 2.**
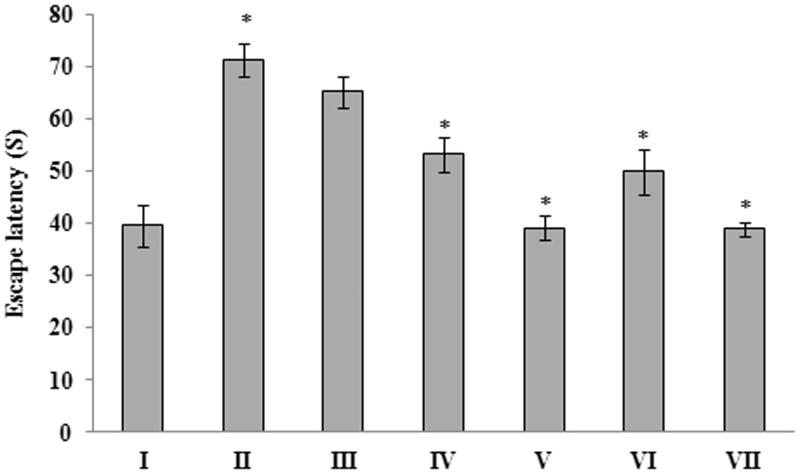
Protective effect of *G. acerosa* benzene extract on Aβ 25–35-induced memory impairment by water-maze test. *p < 0.05 [Comparisons were made between groups II (Aβ 25–35 peptide treated) Vs I (CMC treated) & III (Aβ 25–35 peptide +200 mg/kg of extract in CMC), IV (Aβ 25–35 peptide +400 mg/kg of extract in CMC), V (400 mg/kg bw of extract), VI (Aβ 25–35 peptide + donepezil), VII (1 mg/kg bw of donepezil) Vs II (Aβ 25–35 peptide treated)].

The effect of *G. acerosa* benzene extract on memory improvement was analyzed through water-maze test. All the animals were treated with *G. acerosa* benzene extract for 21 days followed by ICV administration. The animals were trained (to locate the platform) in three trials per day for 2 days. The experiment was performed three days after ICV administration of Aβ 25–35. Their spatial learning scores were recorded as escape latency in seconds. The results of the experiment suggests that, a significant (*p <* 0.05) increase (71.33 ± 3.14) in escape latency was observed in Aβ 25–35 peptide-treated group, when compared to control (39.66 ± 4.03). Treatment with 200 mg/kg and 400 mg/kg of extract exhibited a significant decrease in the escape latency (*p <* 0.05), when compared to peptide treated group (Figure 2). Treatment with the standard drug, donepezil also caused a significant decrease (*p <* 0.05) in escape latency, when compared to the peptide-treated group.

#### Assessment of protective effect of *G. acerosa* on Aβ 25–35-induced memory impairment by step-down inhibitory avoidance test

**Figure 3.**
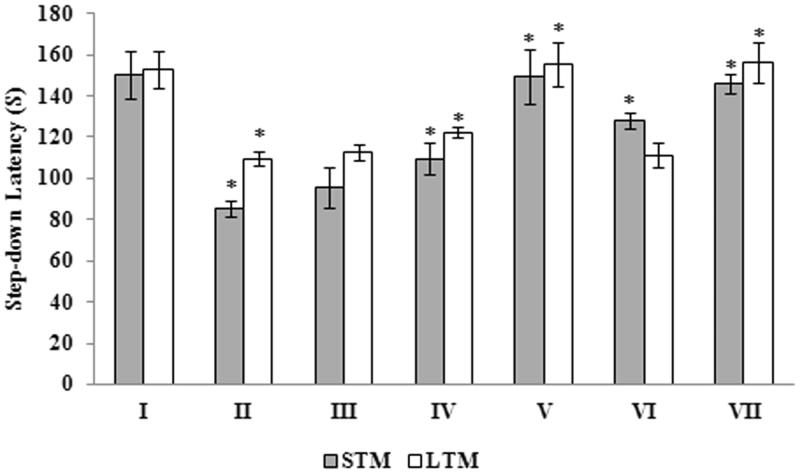
Effect of *G. acerosa* benzene extract on Aβ 25–35-induced memory impairment in mice by step-down inhibitory avoidance test. STM: short term memory; LTM: long term memory. *p < 0.05 [Comparisons were made between groups II (Aβ 25–35 peptide treated) Vs I (CMC treated) & III (Aβ 25–35 peptide +200 mg/kg of extract in CMC), IV (Aβ 25–35 peptide +400 mg/kg of extract in CMC), V (400 mg/kg bw of extract), VI (Aβ 25–35 peptide + donepezil), VII (1 mg/kg bw of donepezil) Vs II (Aβ 25–35 peptide treated)].

The experiments were performed in mice seven days after administration of Aβ 25–35. Treatment with Aβ 25–35 resulted in significant (*p <* 0.05) decrease in STM (85.33 ± 3.55) and LTM (109.5 ± 3.61), when compared to the control animals (150.33 ± 11.70 and 152.5 ± 8.96 for STM and LTM respectively) (Figure 3). Interestingly, treatment with *G. acerosa* benzene extract results in a significant (*p <* 0.05) increase in both STM and LTM which was verified by the increase in step-down latency. The standard drug Donepezil also increases the step-down latency significantly (*p <* 0.05) in the peptide treated animals. Hence, the results of the behavioural assays suggest that *G. acerosa* benzene extract significantly improves both STM and LTM, which was otherwise impaired due to the administration of Aβ 25–35.

### Effect of *G. acerosa* benzene extract on Aβ 25–35-induced alteration in the levels of antioxidant enzymes in mice

**Figure 4.**
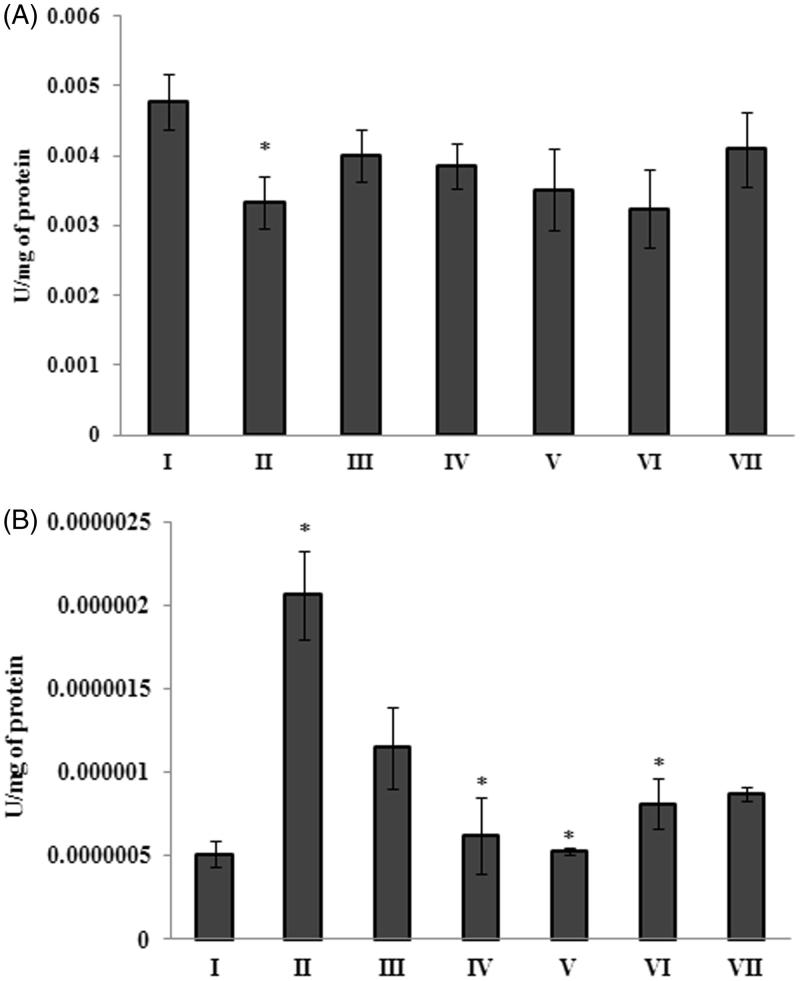
*Gelidiella acerosa* benzene extract restores the alteration in the level of catalase (A) and SOD (B) activity in mice treated with Aβ 25–35. *p < 0.05 [Comparisons were made between groups II (Aβ 25–35 peptide treated) Vs I (CMC treated) & III (Aβ 25–35 peptide +200 mg/kg of extract in CMC), IV (Aβ 25–35 peptide +400 mg/kg of extract in CMC), V (400 mg/kgbw of extract), VI (Aβ 25–35 peptide + donepezil), VII (1 mg/kg bw of donepezil) Vs II (Aβ 25–35 peptide treated)].

Treatment with Aβ 25–35 significantly (*p <* 0.05) reduced the catalase activity (0.0033 ± 0.00037 U/mg of protein) in peptide treated group (Group II), when compared to the control group (0.00477 ± 0.0004 U/mg of protein) (Group I) (Figure 4(A)). This decrease in catalase levels might be due to the increase in the production of peroxides in the brain of peptide-treated animals. Interestingly, the level of catalase was increased (0.0040 ± 0.00038 U/mg of protein) in the extract treated group (200 mg/mL) as that of control group. Animals (treated with Aβ 25–35) treated with Donepezil did not show any effect on catalase activity. Similarly, the effect of *G. acerosa* benzene extract on the levels of SOD was evaluated. The results suggest that Aβ 25–35 induces the level of SOD significantly (*p <* 0.05) in peptide-treated group (Group II). This increase in SOD activity might be due to the compensatory response, in order to scavenge Aβ 25–35-induced production of superoxides. Interestingly, the level of SOD was restored to normal levels as that of control, in the extract treated group (Group III and IV) (Figure 4(B)), which might be due to the radical scavenging potential of *G. acerosa* benzene extract.

**Figure 5.**
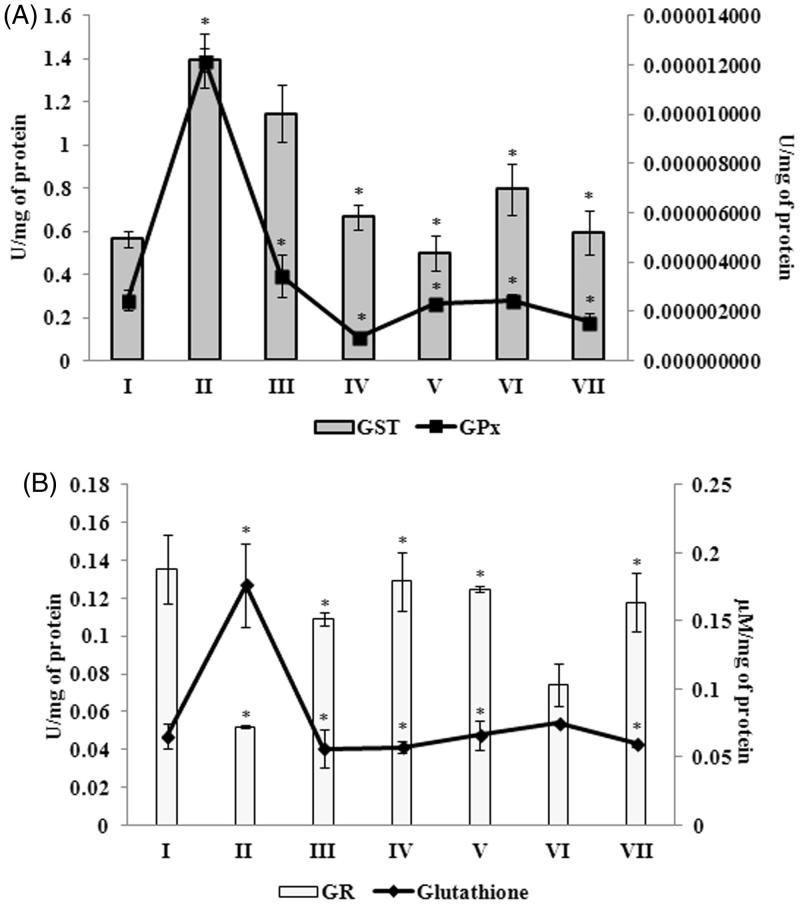
Effect of *G. acerosa* benzene extract on Aβ 25–35 induced alteration in the level of glutathione-*S*-transferase (GST) and glutathione peroxidase (GPx) (A), glutathione reductase (GR) and glutathione (B). *p < 0.05 [Comparisons were made between groups II (Aβ 25–35 peptide treated) Vs I (CMC treated) & III (Aβ 25–35 peptide +200 mg/kg of extract in CMC), IV (Aβ 25–35 peptide +400 mg/kg of extract in CMC), V (400 mg/kg bw of extract), VI (Aβ 25–35 peptide + donepezil), VII (1 mg/kg bw of donepezil) Vs II (Aβ 25–35 peptide treated)].

**Figure 6.**
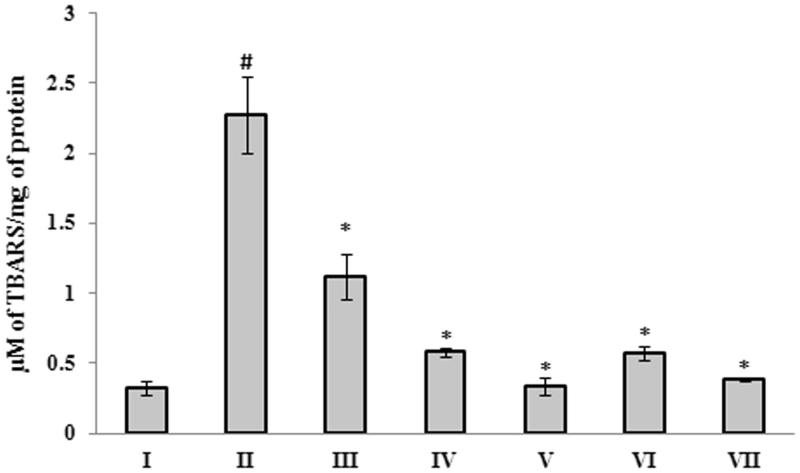
Effect of *G. acerosa* benzene extract on Aβ 25–35-induced lipid peroxidation in brain. The values are expressed as Mean ± SD. *p < 0.05 [Comparisons were made between groups II (Aβ 25–35 peptide treated) Vs I (CMC treated) & III (Aβ 25–35 peptide +200 mg/kg of extract in CMC), IV (Aβ 25–35 peptide +400 mg/kg of extract in CMC), V (400 mg/kg bw of extract), VI (Aβ 25–35 peptide + donepezil), VII (1 mg/kg bw of donepezil) Vs II (Aβ 25–35 peptide treated)].

**Figure 7.**
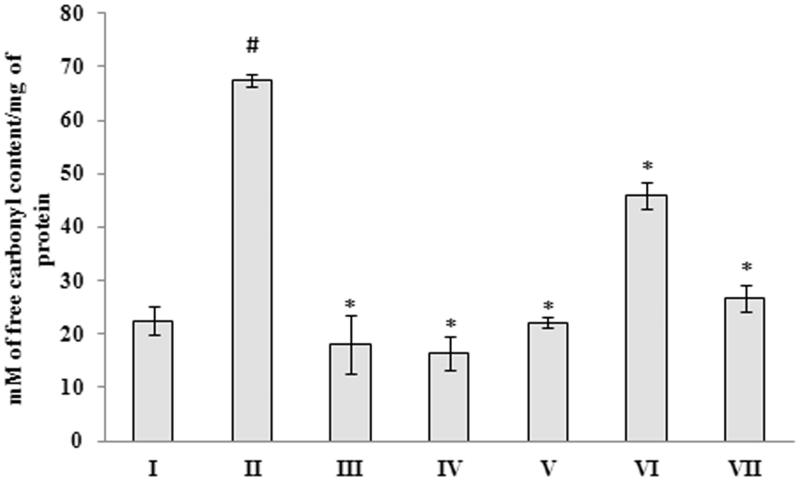
Effect of *G. acerosa* benzene extract on Aβ 25–35-induced protein carbonyl content production in mice brain. The values are expressed as Mean ± SD. *p < 0.05 [Comparisons were made between groups II (Aβ 25–35 peptide treated) Vs I (CMC treated) & III (Aβ 25–35 peptide +200 mg/kg of extract in CMC), IV (Aβ 25–35 peptide +400 mg/kg of extract in CMC), V (400 mg/kg bw of extract), VI (Aβ 25–35 peptide + donepezil), VII (1 mg/kg bw of donepezil) Vs II (Aβ 25–35 peptide treated)].

In addition to catalase and SOD levels, the alteration in the levels of other important antioxidant enzymes such as glutathione-*S*-transferase, glutathione peroxidase and glutathione was also analyzed. The results show that, a significant (*p <* 0.05) increase in glutathione-*S*-transferase, glutathione peroxidase and glutathione activity was observed in Aβ 25–35 peptide-treated group (1.39 ± 0.054 U/mg of protein; 0.000012 ± 1.12E-06 U/mg of protein and 0.176 ± 0.030 μM/mg of protein, respectively), when compared to control group (0.56 ± 0.038 U/mg of protein; 2.46E-06 ± 4.154E-07 U/mg of protein and 0.06 ± 0.009 μM/mg of protein, respectively) (Figure 5(A,B)). Treatment with *G. acerosa* benzene extract restores the level of these antioxidants towards the normal levels, which suggests that benzene extract, through its antioxidant potential reduces the Aβ peptide-induced alteration in the level of antioxidant enzymes. The level of glutathione reductase was decreased significantly (*p <* 0.05) upon treatment with Aβ 25–35 (0.052 ± 0.00055 U/mg of protein), when compared to the control group (0.1353 ± 0.0179 U/mg of protein). Treatment with benzene extract restores the levels towards normal in Aβ-treated mice (Figure 5(B)). Hence, the above results suggest that the extract possess excellent antioxidant potential, as it restores the alteration (induced by Aβ 25–35) in the level of endogenous antioxidant enzymes in mice. The effect of *G. acerosa* benzene extract on the levels of peroxidation of lipids was evaluated in Aβ 25–35-treated mice brain tissue homogenate. A significant increase (*p <* 0.05) in the level of TBARS (2.27 ± 0.27 μM TBARS/mg of protein) was observed in brain of Aβ 25–35 injected mice, when compared to control group (0.323 ± 0.049 μM TBARS/mg of protein) (Figure 6). Treatment with 400 mg of *G. acerosa* benzene extract (Group IV) resulted in a significant decrease (*p <* 0.05) in the level of TBARS (0.58 ± 0.036 μM), which suggests that *G. acerosa* prevents Aβ 25–35-mediated peroxidation of lipids. In order to measure the level of protein oxidation, the amount of protein carbonyls formed were determined. The results showed that a significant increase (*p <* 0.05) in the level of protein carbonyls (67.48 ± 1.11 mM/mg of protein) was observed in the brains of Aβ 25–35-treated mice, when compared to control group (22.53 ± 2.71 mM/mg of protein) (Figure 7). Treatment with *G. acerosa* benzene extract prevented Aβ-mediated protein carbonyl content production, which was verified by the amount of protein carbonyls formed in the extract treated group. Hence, the results suggest that *G. acerosa* benzene extract protects the macromolecules such as lipids and proteins from Aβ 25–35-mediated oxidation.

### Effect of *G. acerosa* benzene extract on Aβ 25–35-induced increase in cholinesterase activity

**Figure 8.**
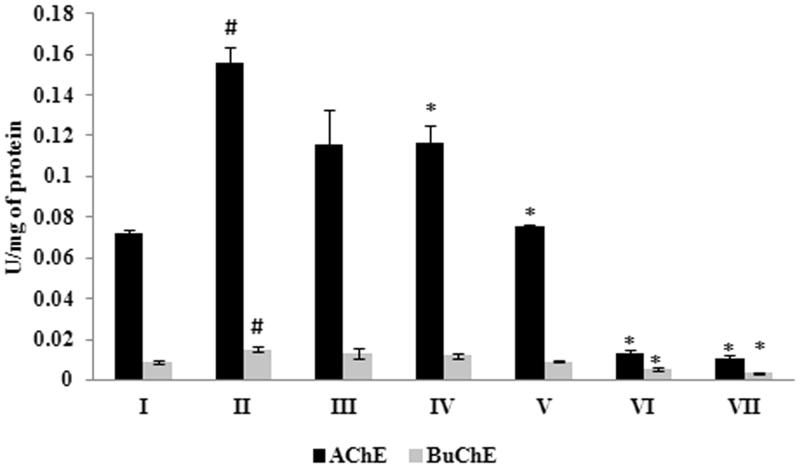
Inhibitory effect of *G. acerosa* benzene extract on Aβ 25–35 induced increase in acetylcholinesterase (AChE) and butyrylcholinesterase (BuChE) activities in mice brain tissue homogenate. The values are expressed as Mean ± SD. *p < 0.05 [Comparisons were made between groups II (Aβ 25–35 peptide treated) Vs I (CMC treated) & III (Aβ 25–35 peptide +200 mg/kg of extract in CMC), IV (Aβ 25–35 peptide +400 mg/kg of extract in CMC), V (400 mg/kg bw of extract), VI (Aβ 25–35 peptide + donepezil), VII (1 mg/kg bw of donepezil) Vs II (Aβ 25–35 peptide treated)].

Since the cholinergic deficit has been greatly implicated in the pathogenesis of AD, the present study involves the evaluation of inhibitory effect of *G. acerosa* benzene extract on Aβ 25–35-induced increase in cholinesterase activities (both AChE and BuChE). The results of the study suggest that Aβ 25–35 induces the level of AChE and BuChE in mice brain (0.155 ± 0.007 U/mg of protein and 0.015 ± 0.0012 U/mg of protein, respectively), significantly (*p <* 0.05) when compared to the control group (0.072 ± 0.0013 U/mg of protein and 0.008 ± 0.0009 U/mg of protein for AChE and BuChE, respectively). *Gelidiella acerosa* benzene extract (at 400 mg concentration) treatment (Group IV) resulted in a significant reduction in the level of these cholinesterases (in peptide treated mice), where the enzyme activity was 0.116 ± 0.0088 U/mg of protein and 0.011 ± 0.0014 U/mg of protein for AChE and BuChE, respectively (Figure 8). Hence, the results suggest that the benzene extract of *G. acerosa* protects the mice brain from cholinergic deficit through the inhibition of ChEs.

### Effect of *G. acerosa* benzene extract on β-secretase activity

**Figure 9.**
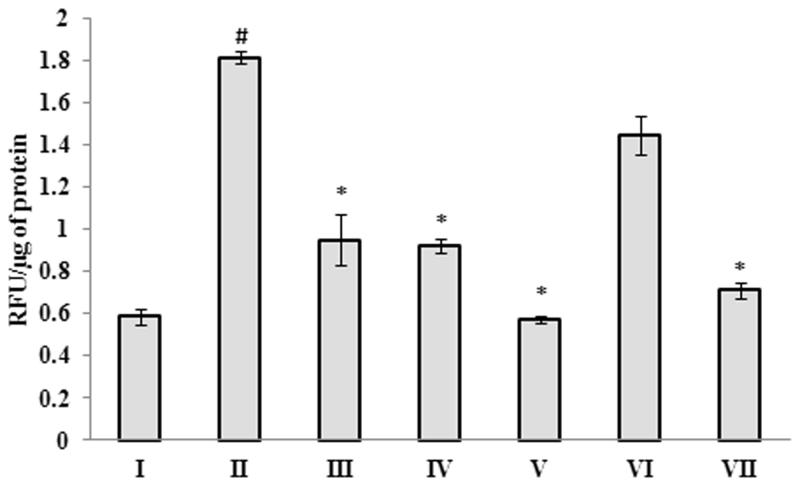
Inhibitory effect of *G. acerosa* benzene extract on Aβ 25–35 induced increase in β-secretase activity in mice brain tissue homogenate. The values are expressed as Mean ± SD. *p < 0.05 [Comparisons were made between groups II (Aβ 25–35 peptide treated) Vs I (CMC treated) & III (Aβ 25–35 peptide +200 mg/kg of extract in CMC), IV (Aβ 25–35 peptide +400 mg/kg of extract in CMC), V (400 mg/kg bw of extract), VI (Aβ 25–35 peptide + donepezil), VII (1 mg/kg bw of donepezil) Vs II (Aβ 25–35 peptide treated)].

The results of the β-secretase assay demonstrated that Aβ 25–35 injection causes significant (*p <* 0.05) increase in the activity of β-secretase (1.817 ± 0.028 RFU/μg of protein), when compared to the control group (0.586 ± 0.035 RFU/μg of protein) (Figure 9). Treatment with *G. acerosa* benzene extract (400 mg) reduced the peptide induced β-secretase activity significantly (*p <* 0.05) with the RFU values of 0.9233 ± 0.029 RFU/μg of protein.

### Protective effect of G. acerosa benzene extract on monoamine oxidase activity

**Figure 10.**
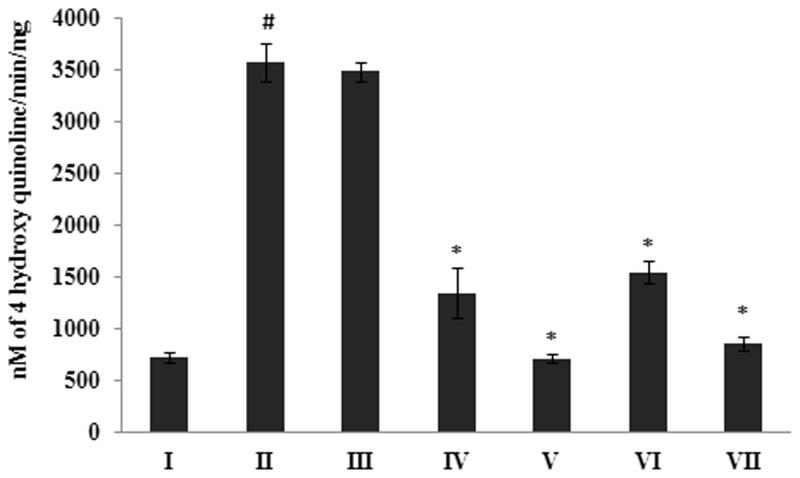
Inhibitory effect of *G. acerosa* benzene extract on Aβ 25–35 induced increase in monoamine oxidase activity in mice brain tissue homogenate. The values are expressed as Mean ± SD. *p < 0.05 [Comparisons were made between groups II (Aβ 25–35 peptide treated) Vs I (CMC treated) & III (Aβ 25–35 peptide +200 mg/kg of extract in CMC), IV (Aβ 25–35 peptide +400 mg/kg of extract in CMC), V (400 mg/kg bw of extract), VI (Aβ 25–35 peptide + donepezil), VII (1 mg/kg bw of donepezil) Vs II (Aβ 25–35 peptide treated)].

The results of the MAO assay showed that, a significant increase (*p <* 0.05) in the levels of MAO-B was observed in peptide treated mice (3572.47 ± 183.47 nM of 4-hydroxy quinoline/min/mg) when compared to the control group (724.28 ± 46.59 nM of 4-hydroxy quinoline/min/mg) (Figure 10). Interestingly, treatment with *G. acerosa* benzene extract (400 mg) reduced the activity of MAO-B (1346.75 ± 239.00 nM of 4-hydroxy quinoline/min/mg) in peptide injected mice significantly (*p <* 0.05).

### Evaluation of anti-apoptotic potential of *G. acerosa* benzene extract on Aβ 25–35-injected mice

**Figure 11.**
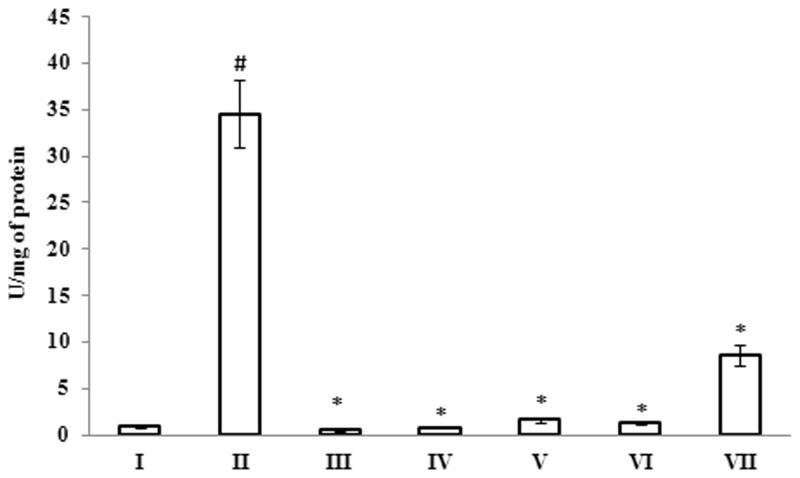
Inhibitory effect of *G. acerosa* benzene extract on Aβ 25–35 induced increase in caspase-3 activity in mice brain tissue homogenate. The values are expressed as Mean ± SD. *p < 0.05 [Comparisons were made between groups II (Aβ 25–35 peptide treated) Vs I (CMC treated) & III (Aβ 25–35 peptide +200 mg/kg of extract in CMC), IV (Aβ 25–35 peptide +400 mg/kg of extract in CMC), V (400 mg/kg bw of extract), VI (Aβ 25–35 peptide + donepezil), VII (1 mg/kg bw of donepezil) Vs II (Aβ 25–35 peptide treated)].

**Figure 12.**
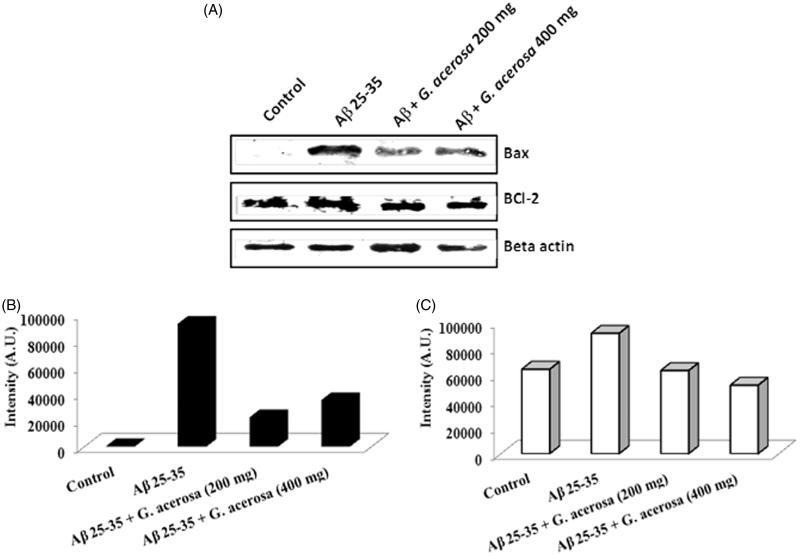
Representative western blot images showing the effect of *G. acerosa* benzene extract on the expression levels of Bax and BCl-2 in mice brain tissue homogenates (A). Quantified results of the expression pattern of Bax (B) and BCl-2 (C).

The results of caspase-3 assay demonstrated that mice treated with Aβ 25–35 causes a significant (*p <* 0.05) increase in caspase-3 activity (34.52 ± 3.62 mM of PNA/min/mg of protein), when compared to untreated control group (0.86 ± 0.14 mM of PNA/min/mg of protein). This increase in the level of caspase-3 activity was reverted to normal levels upon treatment with *G. acerosa* benzene extract (0.81 ± 0.12 mM of PNA/min/mg of protein) (Figure 11). In addition to caspase-3 activity, the anti-apoptotic effect of *G. acerosa* was also assessed by measuring the expression of pro-apoptotic (Bax) and anti-apoptotic proteins (BCl-2) by western blot analysis. The results show that Bax protein expression was increased in Aβ 25–35 peptide group, which suggests that the peptide promotes or induces neuronal death through the process of apoptosis. *Gelidiella acerosa* benzene extract at the concentration of 200 mg and 400 mg reduced the expression level of Bax protein (Figure 12). In addition to that, the expression pattern of BCl-2 was also assessed and the results suggest that treatment with Aβ 25–35 induces the expression of BCl-2, which could be a compensatory response of the cellular machinery in order to prevent the apoptosis mediated cell death. However, *G. acerosa* benzene extract did not affect the expression pattern of BCl-2, which suggests that the extract exerts its anti-apoptotic activity only by down-regulating the expression level of pro-apoptotic protein (Bax).

### Acute toxicity studies of *G. acerosa*

Acute toxicity study of a test substance is employed as an excellent tool for assessing its toxicity risk in human health and environment (Chandrasekaran et al. 2009). The results of acute toxicity studies indicate that *G. acerosa* did not cause any adverse behavioral changes or mortality upon treatment with 2000 mg/kg bw. At the end of the study, all the animals were sacrificed and the organs were collected and analyzed for changes in the organ weight. No significant changes were observed in the weight of organs upon treatment with *G. acerosa* benzene extract (Table 2).

### Haematological and biochemical parameters

Blood is an important index of physiological and pathological condition in animals and human beings. Upon ingestion of the plant constituents, the normal ranges of these parameters may tend to alter and therefore analyzing the haematological parameters has become crucial in toxicity testing (Adeneye et al. 2006). The effect of *G. acerosa* benzene extract on blood constituents were verified by analyzing the haematological and biochemical parameters. Table 3 illustrates the haematological parameters of mice treated with 2000 mg/kg bw of *G. acerosa* benzene extract (acute toxicity study). Minor changes such as increase in the percentage of eosinophil and decrease in the percentage of neutrophils, MCV and monocytes were observed upon treatment with *G. acerosa* benzene extract. However, the changes were not significant in all the parameters analyzed, between control and treated groups. The results of biochemical parameters suggest that a slight decrease in the level of blood sugar and an increase (*p <* 0.05) in the level of sodium were observed upon treatment with *G. acerosa* benzene extract (Table 4). It should be noted that there is no significant change (*p <* 0.05) observed in all the other parameters evaluated.

**Table 2.** Organ weight of mice treated with *G. acerosa* benzene extract (2000 mg/kg of BW).

Organ	Control^a^	*G. acerosa* (2000 mg/Kg BW of mice)^a^
Kidneys^b^	1.433 ± 0.3512	1.233 ± 0.152
Liver^b^	5.167 ± 0.3512	5.4 ± 0.26
Heart^b^	0.4333 ± 0.05774	0.433 ± 0.115
Lungs^b^	0.5667 ± 0.05774	0.623 ± 0.351
Spleen^b^	0.5833 ± 0.03512	0.536 ± 0.0152
Pancreas^b^	0.3667 ± 0.05774	0.233 ± 0.057
Brain^b^	0.48 ± 0.02	0.4567 ± 0.047
Ovary^b^	0.26 ± 0.03464	0.2867 ± 0.030

a Results were expressed as Mean ± S.D.

b g/100 g of BW.

**Table 3.** Haematological profile of mice treated with *G. acerosa* benzene extract (2000 mg/kg of BW).

Parameters	Control^a^	*Gelidiella acerosa* (2000 mg/Kg BW of mice)^a^
Total RBC count (1 × 10^6^ μL)	7.557 ± 0.3636	7.6 ± 0.26
Total WBC count (1 × 10^3^ μL)	5.793 ± 0.1607	5.2 ± 0.305
Platelet count (1 × 10^3^ μL)	179 ± 6.245	174 ± 4
Packed cell volume (%)	41 ± 3	42.67 ± 3.055
HB (g/dL)	12.4 ± 0.8	11.37 ± 0.15
MCV (fl)	47.33 ± 5.686	43.67 ± 2.0
MCH (pg)	6.867 ± 0.2517	6.33 ± 0.25
Neutrophils (%)	69.67 ± 3.215	66 ± 2.64
Lymphocytes (%)	77.33 ± 5.686	76.67 ± 2.08
Eosinophils (%)	0.3333 ± 0.5774	0.666 ± 0.577
Monocytes (%)	1.667 ± 0.5774	1.33 ± 0.577

a Results were expressed as Mean ± S.D.

### Histopathological analysis

**Figure 13.**
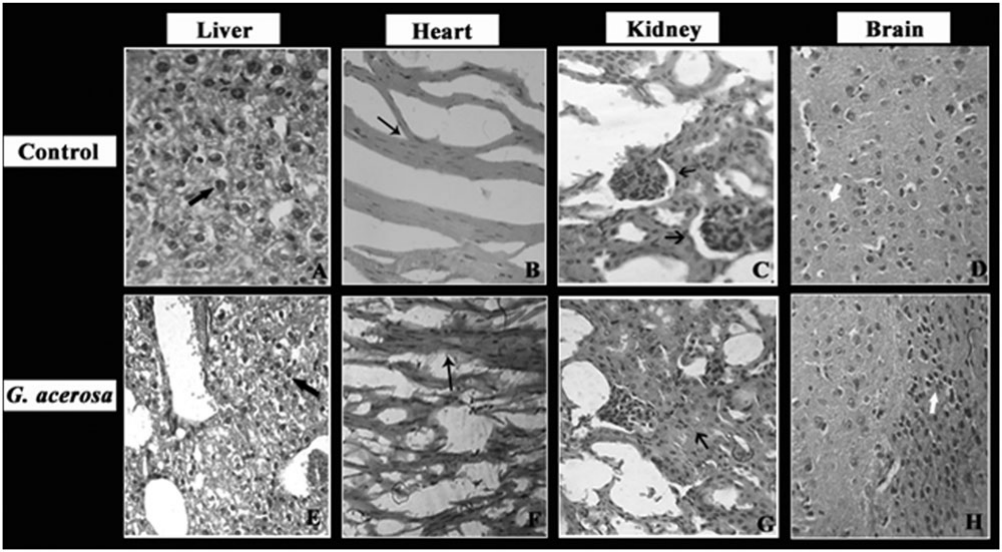
Histopathological analysis of the effect of *G. acerosa* on mice treated with 2000 mg/kg bw. A, B, C & D – Control group; E, F, G, H – *G. acerosa*-treated group. A&E- 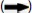 Liver section showing normal hepatocyte architecture without any necrosis. B&F- 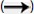 Heart muscles showing intact fibre alignment with normal tissue integrity. C&G- 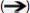 Kidney sections showing normal tubules and interstitium. D&H- 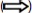 Brain section with normal marginal alignment and interneuronal space.

Figure 13 illustrates the results of histopathological analysis of control and treated groups of acute toxicity study. Generally, the toxic constituents present in the plants and other foreign substances get accumulated in liver which are further subjected to detoxification. Hence, analyzing the architecture of liver tissues is essential (Adeneye et al. 2006). Histopathology of *G. acerosa*-treated mice liver shows that the hepatocyte architecture was normal and sign of necrosis was not observed. Moreover, the liver parenchymal cells were normal both in the control and treated groups. In case of heart tissues, the fibre alignment and cardiac muscles were normal. In addition to that, tissue integrity of heart was also unaltered in the seaweed-treated mice. Histopathology of kidney and brain reveal that tubules and interstitium (in kidney) and marginal alignment and interneuronal space (in brain) were found to be normal in the mice treated with *G. acerosa* benzene extract. Hence, the results suggest that *G. acerosa* benzene extract did not cause any adverse pathological effects during the short-term treatment in mice.

### Sub- acute toxicity studies

The results of sub-acute toxicity studies show that treatment with 200 and 400 mg/kg bw of *G. acerosa* benzene extract (for 28 days) did not cause adverse changes in behaviour or mortality. Parameters like body weight, body tone, salivation, urination, rearing, defecation, locomotion were found to be normal throughout the study period (results not shown). At the end of the study, all the animals were sacrificed and the organs were collected and weighed. No significant changes were observed in the organ weight between the control and treated groups (Table 5).

**Table 4.** Biochemical profile of mice treated with *G. acerosa* benzene extract (2000 mg/kg of BW).

Parameters	Control^a^	*Gelidiella acerosa* (2000 mg/Kg BW of mice)^a^
Blood sugar (mg/dL)	78.33 ± 4.163	71 ± 2
BUN (mg/dL)	24.33 ± 3.055	24.33 ± 2.08
Serum creatinine (mg/dL)	0.8467 ± 0.0602	0.76 ± 0.12
Alkaline phosphatase (U)	100.7 ± 3.512	99.67 ± 3.055
Serum total protein (g/dL)	5.4 ± 0.9644	5.16 ± 0.230
Serum albumin (g/dL)	2.967 ± 0.2082	2.4 ± 0.2
SGOT (AST) (IU/mL)	54 ± 3.606	52.33 ± 3.215
SGPT (ALT) (IU/L)	60 ± 2.646	64 ± 1
Calcium (mmol/L)	6.933 ± 0.3786	7.433 ± 0.30
Sodium (mmol/L)	137.7 ± 4.509	154.3 ± 3.055*
Potassium (mmol/L)	5.267 ± 0.2082	5.4 ± 0.45
Bilirubin total (mg/dL)	0.7 ± 0.1732	0.733 ± 0.115
Serum total cholesterol (mg/dL)	85 ± 4.583	87.33 ± 1.52
Serum triglycerides level (mg/dL)	54.67 ± 3.512	53.83 ± 2.079
Serum HDL cholesterol (mg/dL)	66.67 ± 5.859	64 ± 1.732
Serum LDL cholesterol (mg/dL)	12.33 ± 3.215	11 ± 1
Serum VLDL cholesterol (mg/dL)	10.2 ± 1.908	10.3 ± 0.984

a Results were expressed as Mean ± S.D. *p < 0.05.

### Haematological and biochemical parameters

The haematological profile of mice treated with *G. acerosa* benzene extract (in sub-acute toxicity study) suggest that no significant changes (*p <* 0.05) were observed in all the parameters evaluated (Table 6). The results of biochemical profile of sub-acute toxicity were analyzed and the results suggest a mild increase in the level of blood sugar and SGOT and a significant increase (*p <* 0.05) in sodium (Table 7). All the other parameters did not show any significant changes, when compared to the control group.

**Table 5.** Organ weight of mice treated with *G. acerosa* benzene extracts (200 and 400 mg/kg of BW).

Organ	Control^a^	*Gelidiella acerosa* 200 mg/kg of BW^a^	*Gelidiella acerosa* 400 mg/kg of BW^a^
Kidneys^b^	1.383 ± 0.132	1.203 ± 0.07	1.533 ± 0.103
Liver^b^	5.6 ± 0.229	5.6 ± 0.27	5.65 ± 0.372
Heart^b^	0.483 ± 0.147	0.6 ± 0.109	0.633 ± 0.0816
Lungs^b^	0.6533 ± 0.019	0.665 ± 0.025	0.68 ± 0.0275
Spleen^b^	0.553 ± 0.041	0.5683 ± 0.021	0.5733 ± 0.02251
Pancreas^b^	0.2 ± 0.0632	0.166 ± 0.081	0.233 ± 0.051
Brain^b^	0.465 ± 0.019	0.5567 ± 0.022	0.5667 ± 0.0265
Ovary^b^	0.37 ± 0.02	0.34 ± 0.045	0.37 ± 0.0219

a Results were expressed as Mean ± S.D.

b g/100 g of BW.

**Table 6.** Haematological profile of mice treated with *G. acerosa* benzene extract (200 and 400 mg/kg of BW).

Parameters	Control^a^	200 mg/kg of BW^a^	400 mg/kg of BW^a^
Total RBC count (1 × 10^6^ μL)	7.983 ± 0.42	8.45 ± 0.36	8.38 ± 0.41
Total WBC count (1 × 10^3^ μL)	5.033 ± 0.47	5.81 ± 0.41	6.31 ± 0.56
Platelet count (1 × 10^3^ μL)	178.8 ± 3.65	177 ± 5.83	177.2 ± 5.94
Packed cell volume (%)	44.17 ± 3.43	46 ± 1.89	46.5 ± 2.88
HB (g/dL)	11.55 ± 0.187	11.55 ± 0.30	11.85 ± 0.36
MCV (fl)	46.67 ± 2.5	48.5 ± 2.4	46.5 ± 2.0
MCH (pg)	6.48 ± 0.27	6.76 ± 0.25	6.833 ± 0.12
Neutrophils (%)	67.5 ± 2.07	69 ± 1.897	70.17 ± 5.41
Lymphocytes (%)	75.83 ± 2.85	75.17 ± 3.18	77 ± 2.75
Eosinophils (%)	0.333 ± 0.416	0.666 ± 0.526	0.67 ± 0.306
Monocytes (%)	1.167 ± 0.408	1.57 ± 0.547	1.333 ± 0.56
Basophils (%)	0.166 ± 0.408	0.333 ± 0.051	0.31 ± 0.16

a Results were expressed as Mean ± S.D.

**Table 7.** Biochemical profile of mice treated with *G. acerosa* benzene extract (200 and 400 mg/kg of BW).

Parameters	Control^a^	200 mg/kg of BW^a^	400 mg/kg of BW^a^
Blood sugar (mg/dL)	74.5 ± 5.5	82.83 ± 5.49	85.17 ± 2.78
BUN (mg/dL)	27.3 ± 2.42	26.5 ± 2.81	26.83 ± 2.63
Serum creatinine (mg/dL)	0.8833 ± 0.39	0.901 ± 0.070	0.931 ± 0.64
Alkaline phosphatase (U)	104 ± 4	102.3 ± 1.966	101.2 ± 3.54
Serum total protein (g/dL)	5.317 ± 0.174	6.117 ± 0.523	6.133 ± 0.54
Serum albumin (g/dL)	2.65 ± 0.18	3.05 ± 0.295	3.4117 ± 0.343
SGOT (AST) (IU/mL)	60.83 ± 4.16	62.67 ± 3.882	67.68 ± 3.50
SGPT (ALT) (IU/L)	65.33 ± 2.50	66 ± 3.28	66.33 ± 2.94
Calcium (mmol/L)	7.25 ± 0.48	7.56 ± 0.265	7.68 ± 0.14
Sodium (mmol/L)	152.7 ± 2.066	156.3 ± 2.33	163.5 ± 3.61*
Potassium (mmol/L)	5.667 ± 0.265	5.6 ± 3.033	5.917 ± 0.098
Bilirubin total (mg/dL)	0.8 ± 0.167	0.6167 ± 0.1169	0.8 ± 0.154
Serum total cholesterol (mg/dL)	85.17 ± 2.137	85 ± 4.817	83.33 ± 2.25
Serum triglycerides level (mg/dL)	55 ± 2.28	55.17 ± 3.25	54.5 ± 3.98
Serum HDL cholesterol (mg/dL)	65.5 ± 2.58	65.67 ± 3.14	67.17 ± 2.99
Serum LDL cholesterol (mg/dL)	10.33 ± 1.21	13.33 ± 2.33	12 ± 1.89
Serum VLDL cholesterol (mg/dL)	10.67 ± 1.211	11 ± 2	10.83 ± 1.83

a Results were expressed as Mean ± S.D. *p < 0.05.

### Histopathological analysis

**Figure 14.**
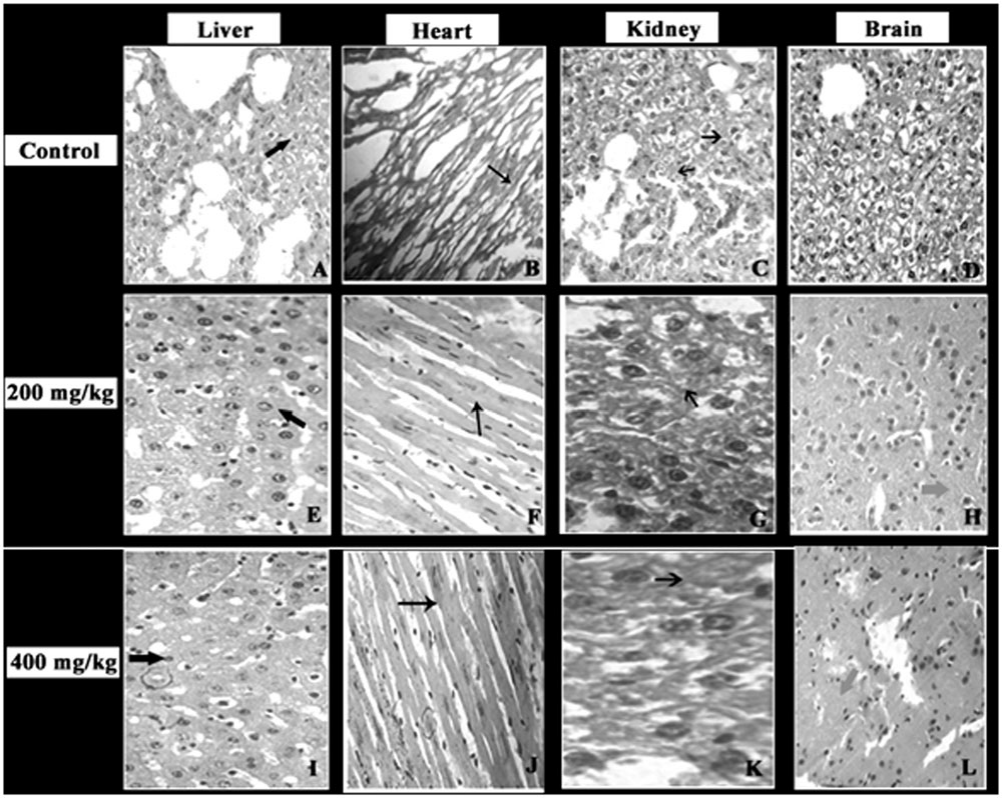
Histopathological analysis of the effect of *G. acerosa* on mice treated with 200 and 400 mg/kg bw. A, B, C, & D – Control group; E,F,G&H – 200 mg/kg bw *G. acerosa*-treated group, IJKL - 400 mg/kg bw *G. acerosa* treated group. A,E & I 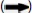 - Liver section showing normal hepatocellular arrangement with intact membrane. B,F & J 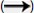 - Heart section showing normal fibre integrity and cell alignment. C, G & K 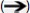 - Kidney section showing normal interstitium. D, H & L 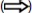 – Brain section shows normal interneuronal space with normal cellular integrity.

The results of histopathological analysis of sub-acute toxicity studies are illustrated in Figure 14. Microscopic images of *G. acerosa*-treated (200 and 400 mg/kg bw) mice liver shows normal hepatocellular arrangement with intact membrane. Histopathology of heart of mice treated with 200 and 400 mg/kg bw shows normal fibre integrity and cell alignment. Kidney sections of mice treated with *G. acerosa* benzene extract shows normal prominent interstitium with no signs of oedema. Histopathology of brain shows normal interneuronal space with well-developed neurons. Also, no signs of inflammation or haemorrhage were observed. Hence, the results of histopathological analysis of all the vital organs suggest that *G. acerosa* did not exhibit any pathological effects in mice treated with *G. acerosa* (both 200 and 400 mg/kg bw).

## Discussion

In the present study, the neuroprotective efficacy of *G. acerosa* benzene extract was evaluated in Aβ 25–35-treated Swiss Albino mice. In order to study the loss of memory by peptide treatment and subsequent protection by the extract, in the present study, the behavioural assays such as Y-maze test, water maze test and step-down inhibitory avoidance test were performed. The results suggest that *G. acerosa* benzene extract improves both LTM and STM of mice, which was otherwise impaired upon treatment with Aβ 25–35. In a similar study led by Myung et al. (2005), demonstrated that the phlorotannins obtained from the brown alga *Eisenia* and *Ecklonia* exhibit noticeable effect in improving the memory of ethanol-treated mice. One of the key components in AD pathological cascade is the oxidative stress. Several modifications such as peroxidation of lipids, protein oxidation and DNA damage has been found to occur as a result of increased accumulation of ROS in the patients of AD brain. Moreover, Aβ peptide-induced oxidative stress has been greatly implicated in AD. The peptide induced toxicity is mediated by free radicals, which causes severe macromolecular damage through the inhibition of antioxidants. Hence, for reducing the ROS-mediated damage in AD, antioxidant therapies particularly, antioxidants from natural source have been proposed for AD treatment (Kim et al. 2011). Therefore, in the present study the effect of *G. acerosa* benzene extract, which has the antioxidant activity, was evaluated on Aβ 25–35-induced alteration in the levels of various endogenous antioxidant defence systems in mice. The results demonstrated that *G. acerosa* benzene extract effectively restores the level of endogenous antioxidants such as catalase, SOD, GSH, GPx, GST and GR in mice administered with Aβ 25–35 peptide. In addition to that, the increase in the amount of lipid peroxidation products (due to peptide treatment) was also reduced significantly as that of control mice. Similarly, Oligonol, which is a polyphenol derived from lychee fruit has been reported to reduce the level of lipid peroxidation in mice treated with Aβ 25–35 peptide (Choi et al. 2014). Apart from lipid peroxidation, oxidation of proteins has been considered as an important factor in aging and age-related neurodegenerative disorders. These oxidative modifications causes altered and nonspecific protein functioning. The protein oxidation can be indexed by measuring the amount of protein carbonyls present in the system. The protein carbonyls are formed from the direct free radical attack on vulnerable amino acid side chains or from the products of glycation, glycoxidation and lipid peroxidation reactions with that of protein. Moreover, the protein carbonyls have also been found to impart neurodegeneration in AD brain (Jhoo et al. 2004). Therefore, in the present study, the amount of protein carbonyls formed due to Aβ-induced toxicity was evaluated. The results suggest that *G. acerosa* benzene extract reduces the amount of protein carbonyls in mice brain treated with Aβ 25–35. Therefore, from the above findings, it is apparent that *G. acerosa* benzene extract exhibit excellent antioxidant capability against Aβ 25–35-induced oxidative stress.

Heretofore, the anti-Alzheimer’s drugs that are approved by FDA targets the most crucial enzyme, which is involved in the neurotransmission is the cholinesterases (AChE and BuChE). Therefore, in the present study, the effect of *G. acerosa* benzene extract on the activity of these ChEs was also analyzed. The results revealed that *G. acerosa* benzene extract reduced the activity of ChEs in Aβ 25–35 peptide-treated mice brain efficiently. The study led by Muralidharan et al. (2010) demonstrated that the ethyl acetate extract of *Morinda citrifolia* L. (Rubiaceae) reduced the AChE activity in mice treated with Aβ 25–35 peptide. Since Aβ peptide plays a crucial role in the onset and progression of AD, the current therapeutic target is to reduce the production of these toxic peptides in CNS of the affected individuals. The suppression of these toxic peptides can be achieved only by inhibiting the activity of the enzyme β-secretase, which is responsible for amyloidogenic processing of APP in AD brain (Lee et al. 2009). Therefore, in the present study, the inhibitory effect of *G. acerosa* benzene extract on β-secretase was evaluated in Aβ 25–35 administered mice. The results suggested that *G. acerosa* benzene extract reduced the activity of β-secretase in peptide-treated mice, which indicates that the extract prevents the generation of Aβ in mice.

Monoamine oxidase in the brain catalyzes the oxidative deamination of biogenic amines and other neurotoxins. Two isozymes are present such as MAO-A and MAO-B, which is distinguished by their differences in substrate and inhibitor selectivity. MAO-B has been found to play a vital role in CNS and peripheral organs. Altered activities of MAO-B is implicated in neurological disorders such as AD, as the oxidation mechanism of MAO-B leads to accumulation of hydrogen peroxide, which in turn causes oxidative stress mediated cytotoxicity. Hence, the development and identification of MAO-B inhibitors are of great interest in the field of drug discovery against AD (Viña et al. 2012). In line with that, the present study involves the evaluation of MAO-B inhibitory activity of *G. acerosa* benzene extract in Aβ 25–35 injected mice and the results suggest that *G. acerosa* benzene extract inhibits MAO-B effectively in peptide-treated mice brain tissue, which shows that the extract prevents the mice from MAO-B-mediated oxidative stress.

The process of programmed cell death or apoptosis plays an important role in the degeneration of neuronal cells, which has been routinely observed in AD brain (Blasko et al. 2000). The AD brain tissues were found to possess deposits of activated caspase-3, which promotes the apoptotic cascade mechanism in neuronal cells (Luo et al. 2002). Hence, the development of compounds with anti-apoptotic potential might be an effective therapeutic strategy in minimizing the apoptosis mediated cell death. In accordance to that, in the present study, the protective effect of *G. acerosa* benzene extract was evaluated against Aβ 25–35-mediated apoptosis in peptide-treated mice. Since caspase-3 has been implicated as an important enzyme involved in apoptosis, the inhibitory activity of *G. acerosa* against caspase-3 enzyme was assessed. The results suggest that *G. acerosa* effectively reduces the activity of caspase-3 in Aβ 25–35-treated mice. To further validate the anti-apoptotic potential of the extract, the expression level of anti-apoptotic protein, BCl-2 and pro-apoptotic protein Bax was analyzed quantitatively. The results show that *G. acerosa* benzene extract reduces the Bax expression in mice treated with Aβ 25–35, which indicates that *G. acerosa* has potent anti-apoptotic efficacy against Aβ induced toxicity. In the case of BCl-2, an increase in the expression was observed, which might be a compensatory response by the cellular machinery in order to prevent apoptosis. Moreover, *G. acerosa* benzene extract did not exhibit any noticeable effect on the expression pattern of the anti-apoptotic protein BCl-2. The results of *in vivo* acute and sub-acute toxicity tests suggest that benzene extract did not exhibit any significant alterations in haematological and biochemical parameters. Moreover, the histopathological analysis of vital organs like liver, heart, kidney and brain revealed that the extract did not cause any adverse pathological effects in mice.

## Conclusion

In the present study, the neuroprotective effect of *G. acerosa* benzene extract was evaluated on Aβ 25–35-treated albino mice. The results of the experiments suggest that *G. acerosa* benzene extract protects the mice brain from Aβ 25–35-mediated cognitive decline and it also restores the peptide-induced alteration in the level of activities of antioxidant enzymes. Moreover, the benzene extract exhibit its protective effect by preventing the cells from macromolecular damage. Reduction in the activities of AChE and BuChE suggests that the extract protects the mice from cholinergic deficit. The extract also possesses inhibitory effect on β-secretase and hence it could be employed as a potential anti-amyloidogenic compound. The reduction in the level of caspase-3 activity and expression pattern of Bax protein suggests that the extract has excellent anti-apoptotic activity. The results of toxicity tests suggest that the *G. acerosa* benzene extract did not cause toxic effects, when evaluated through both acute and sub-acute toxicity tests. Overall, the outcome of the study suggests that *G. acerosa* benzene extract holds multifarious neuroprotective potential against Aβ 25–35-mediated toxicities.
